# No bladder irrigation versus continuous bladder irrigation after HoLEP: a propensity score matching analysis

**DOI:** 10.1186/s12894-023-01187-9

**Published:** 2023-02-17

**Authors:** Yunwu Hao, Degang Chen, Xudong Shen, Yang Chen, Zongyao Hao

**Affiliations:** 1 Department of Urology, The First Affiliated Hospital of Anhui Medical University, Hefei, 230022 Anhui China; 2 Department of Urology, Lu’an Hospital Affiliated of Anhui Medical University, Lu’an, 237000 Anhui China; 3 Institute of Urology, Anhui Medical University, Hefei, 230022 Anhui China; 4 Anhui Province Key Laboratory of Genitourinary Diseases, Anhui Medical University, Hefei, 230022 Anhui China

**Keywords:** No bladder irrigation strategy, Benign prostatic hyperplasia, Holmium laser, Enucleation of the prostate, Surgery

## Abstract

**Purpose:**

In this study, the feasibility of a no bladder irrigation strategy after transurethral holmium laser enucleation of the prostate (HoLEP) for the treatment of benign prostatic hyperplasia (BPH) was studied.

**Methods:**

From August 2021 to December 2021, the clinical data of 62 patients who received no bladder irrigation after HoLEP (Group A) were studied. The control group contained the clinical data of 150 patients in the same therapy group (from January 2021 to July 2021) who received continuous bladder irrigation after HoLEP (Group B). The baseline was consistent after using the propensity score matching (PSM) method, and the differences between groups were compared. The pre- and postoperative complications, international prostate symptom score (IPSS), quality of life (QOL), maximum urinary flow rate (Qmax), and postvoid residual urine (PVR) of the two groups were compared, accompanied by a follow-up evaluation of surgical effects.

**Results:**

47 pairs of patients were successfully matched by PSM. There was no statistically significant difference in the intraoperative conditions and the incidence of early postoperative complications between the two groups (*P* > 0.05). Before and one month after the surgery, significant differences were also found in the IPSS, QOL, Qmax, and PVR of both groups (*P* < 0.05). Within one month after the surgery, no statistically significant difference was found in IPSS, QOL, Qmax, PVR, or the incidence of early postoperative complications between the two groups (*P* > 0.05).

**Conclusion:**

For appropriately selected patients according to the exclusion criteria, the no bladder irrigation strategy after HoLEP for BPH is safe and effective.

## Introduction

Benign prostatic hyperplasia (BPH) has long been recognized as a common disease affecting the health of elderly individuals [1]. Accompanying BPH development, blockage of the bladder outlet may deteriorate, resulting in urine retention, repeated haematuria, bladder stones, recurrent urinary tract infections, and possibly other relevant severe problems, such as hydrops of the upper urinary tract and renal insufficiency. Transurethral surgery is the most commonly performed procedure for BPH surgery, including transurethral resection of the prostate (TURP), holmium laser enucleation of the prostate (HoLEP), thulium laser enucleation of the prostate (ThuLEP), greenlight laser enucleation of the prostate (GreenLEP), and greenlight laser vapourization of the prostate (photoselective vapourization of the prostate [PVP]) [2]. The TURP technique has several drawbacks, e.g., insufficient excision of the prostate tissue, TUR syndrome, excessive bleeding, and limited prostate volume [3]. In contrast, the HoLEP technique has become one of the most effective alternatives to BPH surgery because of the shorter catheterization and hospital stay, effective haemostasis, and fewer complications [4–6]. Related research has shown that HoLEP is superior to conventional transurethral prostate enucleation techniques [5, 7]. Additionally, it is thought to have the best chance of becoming the gold standard for the treatment of BPH [8].

In terms of BPH surgery, postoperative bleeding is the most significant complication independent of open surgery, TURP and the HoLEP procedure. To overcome this, the main strategy for postoperative bleeding is continuous bladder irrigation to avoid the formation of clots that can block the urinary catheter. At the same time, the urinary catheter can be pulled, and the untreated blood vessel haemorrhage can be squeezed using the urine catheter balloon. With the development of minimally invasive surgery, the blood loss associated with HoLEP surgery has been decreasing. Meanwhile, the prostatic fossa wound may be bloodless after the surgery. Related studies have also shown that the time required after bladder irrigation is decreasing, and in some cases, daytime surgery has been implemented for BPH surgery [9–12]. Therefore, the time of continuous bladder irrigation postoperative has been decreasing and it may not be considered an essential step after HoLEP for BPH surgery.

In this study, a no-bladder irrigation strategy after HoLEP for BPH surgery was studied based on a database containing the clinical data of patients who received no-bladder irrigation after HoLEP for BPH surgery (August 2021 to December 2021, Lu’an Hospital Affiliated of Anhui Medical University). The findings of this study can provide an improved understanding of the no bladder irrigation strategy after HoLEP, which can influence its application in prostate-related minimally invasive surgery.

## Materials and methods

### Patients

**Fig. 1:**
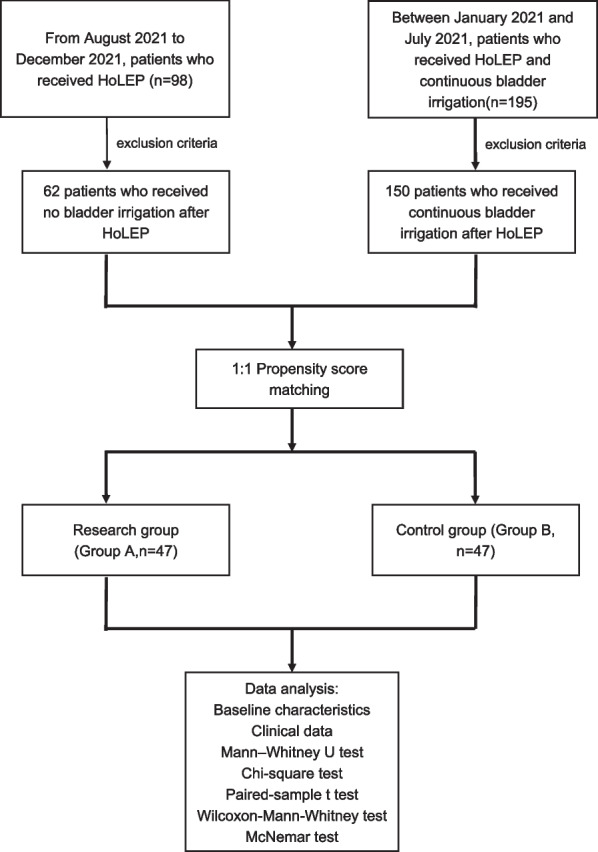
Flowchart of study population

From August 2021 to December 2021, 98 patients received HoLEP for BPH surgery. Based on the exclusion criteria, 62 patients who received no bladder irrigation after the surgery were chosen as the research group (Group A). Between January 2021 and July 2021, 195 patients received HoLEP, among which the clinical data of 150 patients who received continuous bladder irrigation after the surgery were chosen based on the exclusion criteria as the control group (Group B). We performed propensity score matching (PSM) for a total of 47 pairs that were successfully matched after reducing the effect of potential confounders such as age, disease duration, and prostate volume between groups. Indications for surgery were based on Chinese urological disease diagnosis and treatment guidelines on BPH (2019). The design of the study was approved by the Ethics Committee at Lu’an Hospital Affiliated of Anhui Medical University, and all patients gave written and informed consent to participate. This study was performed in accordance with the Declaration of Helsinki and with standards of good clinical practice. Figure 1 presents a flowchart describing the selection of the study population.

Exclusion criteria for two groups of patients included the following: (1) patients with severe organ dysfunction, such as cardiovascular or cerebrovascular disease; (2) patients with a history of malignant tumours or prostate biopsy before the surgery; (3) patients who previously had received TURP or transurethral enucleation of the prostate; and (4) patients whose prostate volume was more than 100 ml.

### Equipment and techniques

The 96 W-SRM-H3B -Ho laser generator, with a 550 µm fibre and a 26 Fr resec-toscope sheath, was used during the HoLEP surgery. The energy settings were set as 2 J, 40 Hz for cutting and 0.8 J, 40 Hz for coagulation. Morcellation was performed with a YSB-III morcellator. The majority of patients received en bloc HoLEP surgery. After performing urethral cystoscopy, an inverted U-shaped laser incision was made anterior to the seminal vesicle mound. The surgeon was able to easily locate the level of the prostate surgical capsule. Along the surgical capsule plane, the middle and bilateral lobes of the prostate are easily separated from the envelope. Second, the urethral mucosal strip between the external sphincter and two lateral lobes was cut by the laser, creating another inverted U-shaped incision. Subsequently, at the 12 o’clock position of the bladder neck, the proximal end of the mucosa was incised to the upper part of the prostatic adenoma, and the depth reached the surgical capsule. Along this level, the middle and bilateral lobes are completely separated. The muscle fibres of the bladder neck are laser dissected to access the bladder lumen as the enucleation is about to reach the bladder neck. This dissection is limited to the surgical capsule. At this stage, both lobes are completely separated from the bladder neck. Then, retrograde separation of the middle lobe along the level of the surgical capsule was performed to reach the bladder neck. The entire prostatic adenoma is then completely pushed into the bladder lumen. Careful coagulation was performed at the prostatic fossa to stop the bleeding. Subsequently, intravesical comminution and extraction were performed by aspiration of the prostate tissue. We used a 20-Fr three-way catheter with 20 ml saline blocked within the bladder. Postoperatively, no bladder irrigation was applied in Group A, and continuous saline bladder irrigation was applied in Group B. Due to the difficulty of enucleating the whole prostatic adenoma (prostate volume > 80 ml), the three-lobe technique was utilized to segment the prostate. All operations were performed by the same surgeon.

### Observation indicator

The preoperative baseline data, surgery time, haemoglobin decline, capsule perforation, catheterization time, and hospital stay of the patients were compared between the two groups. Within one month after the surgery, the urine retention and gross haematuria, haemorrhage, temporary urinary incontinence, urinary tract infection, testicular epididymitis, and other complications of the patients between the two groups were monitored. One month and six months after the surgery, different parameters, including IPSS, QOL, Qmax, and PVR, were compared between the two groups.

### Statistical analysis

The statistical analysis was performed using SPSS 25 (IBM Corp, Armonk, NY, USA). PSM was implemented using the PSM extension procedure in IBM SPSS. The nearest neighbor algorithm was used as the matching method, with the caliper value set to 0.02 and the ratio set to 1:1. Before matching, for the data with a normal distribution, independent sample t tests were used, and for the data with an abnormal distribution, Mann–Whitney U tests were used. Chi-square tests were used to compare the categorical variables. After matching, normally distributed variable data analysed with paired-sample t test. For the data with a nonnormally distributed variable data, Wilcoxon-Mann–Whitney tests were used, and categorical variable data analysed with the McNemar test. *P* < 0.05 was considered statistically significant. All of the experimental data are displayed as the average value ± standard deviation.

## Results

The primary outcome was the difference in baseline characteristics between the two groups before matching (Table 1). We performed PSM for a total of 47 pairs that were matched successfully. Preoperative baseline characteristics such as age, disease duration, anticoagulants, alpha blockers, urogenital complications, prostate volume, PSA, IPSS, QoL, Qmax and PVR were not statistically significant between the two groups (*P* > 0.05, Table 2). There was no significant difference in operative time, resected prostate weight or haemoglobin decrease between the two groups (*P* > 0.05). There were statistically significant differences in IPSS, QoL, Qmax, and PVR between the two groups. One patient in Group A and two patients in Group B underwent prostate capsule puncture during surgery, but no visible bleeding occurred, and no additional treatment needed to be provided. There was no bladder damage or blood transfusion in either group. The differences in catheterization time and hospital stay between the two groups were not statistically significant (*P* > 0.05). Within one month after the surgery, there were no statistically significant differences in early postoperative complications, such as urine retention, gross haematuria, haemorrhage, transitory urinary incontinence, urinary tract infection, testicular epididymitis, and urethral stricture, between the two groups (*P* > 0.05, Table 3). Before and one month after the surgery, there were statistically significant differences in IPSS, QoL, Qmax, and PVR in the two groups (*P* < 0.05, Table 4). At one month and six months after the surgery, there were no statistically significant differences in IPSS, QoL, Qmax, or PVR between the two groups (*P* > 0.05, Table 5).

**Table 1:** Baseline criteria of the two groups before PSM

	Group A	Group B	*P* value
Patients, n	62	150	
Age (year)^a^	70.4 ± 7.0	73.2 ± 6.5	0.027
Disease duration (year)^a^	5.2 ± 2.2	6.6 ± 3.2	0.006
Anticoagulants^b^	13 (21%)	35 (23.3%)	0.708
Alpha blockers^b^	32 (51.6%)	78 (52%)	0.959
5ARIs alone or combined with alpha blockers^b^	22 (35.5%)	58 (38.7%)	0.664
Urogenital complications			
With acute urinary retention^b^	17 (27.4%)	47 (31.3%)	0.572
With gross haematuria^b^	19 (30.6%)	47 (31.3%)	0.992
With bladder stones^b^	12 (21.3%)	32 (21.3%)	0.747
With bladder trabeculae or chambers^b^	33 (53.2%)	81 (54%)	0.918
Prostate volume (ml)^a^	63.4 ± 20.2	69.4 ± 14.9	0.004
IPSS^a^	24.6 ± 4.8	26.2 ± 4.7	0.009
QoL^a^	4.9 ± 0.8	4.7 ± 0.8	0.047
Qmax (ml/s)^a^	6.3 ± 2.4	7.6 ± 2.3	0.001
PVR (ml)^a^	147.2 ± 139.8	135.5 ± 124.3	0.662
PSA (ng/ml)^a^	4.0 ± 2.5	4.0 ± 2.0	0.776

^a^Nonnormally distributed variable data analysed with the Mann‒Whitney U test

^b^Categorical variable data analysed with the chi-square test

**Table 2:** Baseline criteria of the two groups after PSM

	Group A	Group B	*P* value
Patients, n	47	47	
Age (year)^a^	71.7 ± 6.4	72.3 ± 6.8	0.662
Disease duration (year)^b^	5.3 ± 2.2	5.0 ± 1.9	0.501
Anticoagulants^c^	12 (25.5%)	8 (17.0%)	0.503
Alpha blockers^c^	22 (46.8%)	26 (55.3%)	0.532
5ARIs alone or combined with alpha blockers^c^	16 (34.0%)	15 (31.9%)	1.000
Urogenital complications			
With acute urinary retention^c^	13 (27.7%)	14 (29.8%)	1.000
With gross haematuria^c^	16 (34%)	15 (31.9%)	1.000
With bladder stones^c^	7 (14.9%)	8 (17.0%)	1.000
With bladder trabeculae or chambers^c^	27 (57.4%)	25 (53.2%)	0.832
Prostate volume (ml)^b^	65.3 ± 20.6	67.0 ± 10.8	0.369
IPSS^b^	25.3 ± 4.9	25.6 ± 4.7	0.473
Qob^b^	4.8 ± 0.7	4.9 ± 0.8	0.421
Qmax (ml/s)^b^	6.6 ± 2.4	6.7 ± 2.3	0.941
PVR (ml)^b^	148.0 ± 140.7	145.4 ± 130.8	0.820
PSA (ng/ml)^b^	3.9 ± 2.6	3.8 ± 2.0	0.992

^a^Normally distributed variable data analysed with paired-sample t test

^b^Nonnormally distributed variable data analysed with the Wilcoxon-Mann–Whitney test

^c^Categorical variable data analysed with the McNemar test

**Table 3:** Intraoperative conditions and early postoperative complications in the two groups

	Group A	Group B	*P* value
Operative time (min)^a^	77.5 ± 14.4	79.5 ± 17.2	0.558
Resected prostate weight (ml)^b^	48.5 ± 14.6	50.8 ± 11.7	0.249
Haemoglobin decrease (g/dl)^a^	9.9 ± 5.4	11.1 ± 6.2	0.328
Capsular perforation^c^	1 (2.1%)	2 (4.3%)	1.000
Catheterization time (d)^b^	2.4 ± 0.7	2.6 ± 0.6	0.225
Hospital stay (d)^b^	4.3 ± 0.5	4.5 ± 0.6	0.141
Urinary retention (%)^c^	3 (6.4%)	4 (8.5%)	1.000
Postoperative Haematuria (%)^c^	4 (8.5%)	6 (12.8%)	0.727
Haemorrhage (%)^c^	1 (2.1%)	2 (4.3%)	1.000
Transient incontinence (%)^c^	14 (29.8%)	16 (34%)	0.845
Urinary tract infections (%)^c^	3 (6.4%)	5 (10.6%)	0.727
Testicular epididymitis (%)^c^	1 (2.1%)	2 (4.3%)	1.000
Urethral stricture (%)^c^	2 (4.3%)	3 (6.4%)	1.000

^a^Normally distributed variable data analysed with paired-sample t test

^b^Nonnormally distributed variable data analysed with the Wilcoxon-Mann–Whitney test

^c^Categorical variable data analysed with the McNemar test

**Table 4:** IPSS, QoL, Qmax and PVR in the two groups preoperative and 1 month after surgery

	Group A				Group B			
	IPSS^a^	QoL^a^	Qmax (ml/s)^a^	PVR (ml)^a^	IPSS^a^	QoL^a^	Qmax (ml/s)^a^	PVR (ml)^a^
Pre	25.3 ± 4.9	4.8 ± 0.7	6.6 ± 2.4	148.0 ± 140.7	25.6 ± 4.7	4.9 ± 0.8	6.7 ± 2.3	145.4 ± 130.8
Post	8.2 ± 3.2	1.8 ± 1.0	18.8 ± 4.5	6.8 ± 3.9	7.9 ± 3.3	1.9 ± 1.0	19.2 ± 4.1	7.4 ± 2.9
*P* value	< 0.001	< 0.001	< 0.001	< 0.001	< 0.001	< 0.001	< 0.001	< 0.001

^a^Nonnormally distributed variable data analysed with the Wilcoxon–Mann–Whitney test

**Table 5:** Follow-up data 1 month and 6 months after surgery in the two groups

	Group A	Group B	*P* value
1 month after surgery			
IPSS^b^	8.2 ± 3.2	7.9 ± 3.3	0.695
QoL^b^	1.8 ± 1.0	1.9 ± 1.0	0.545
Qmax (ml/s)^b^	18.8 ± 4.5	19.1 ± 4.0	0.533
PVR(ml)^b^	6.8 ± 3.9	7.4 ± 2.9	0.334
6 months after surgery			
IPSS^b^	7.2 ± 3.1	7.1 ± 3.2	0.985
QoL^b^	1.1 ± 0.8	1.3 ± 0.9	0.420
Qmax (ml/s)^a^	19.7 ± 4.2	19.3 ± 3.6	0.624
PVR(ml)^b^	6.6 ± 3.1	6.5 ± 2.9	0.851

^a^Normally distributed variable data analysed with paired-sample t test

^b^Nonnormally distributed variable data analysed with the Wilcoxon

## Discussion

This study compared intraoperative conditions, early postoperative complications, and follow-up data in patients who received no bladder irrigation after HoLEP with continuous bladder irrigation. First, we showed that Group A and Group B had similar outcomes before surgery by PSM. Second, two groups had similar findings in terms of intraoperative conditions and early postoperative complications. However, the workload of healthcare workers is significantly reduced by this method. Meanwhile, the discomfort symptoms of patients during continuous bladder irrigation can also be significantly alleviated. Finally, there were no statistically significant differences in follow-up data 1 month and 6 months after surgery. This indicates that the two groups have similar long-term treatment effects.

The duration of continuous bladder irrigation after HoLEP varies, with the shortest bladder irrigation time being only 2 h [13, 14]. There were also reports of no bladder irrigation after surgery for the treatment of benign prostatic hyperplasia [15]. However, the disadvantage of using robot-assisted simple prostatectomy to treat benign prostatic hyperplasia is that it is not a natural orifice treatment method. Meanwhile, this choice of surgical approach added additional surgical trauma. In our study, we propose that the strategy of no bladder irrigation after after HoLEP is feasible, which simplifies the postoperative treatment steps. After endoscopic enucleation of the prostate, bladder irrigation is used to prevent blood clots from obstructing the urinary catheter but has little haemostatic effect. The theoretical basis of no bladder irrigation after enucleation of the prostate are: (1) there is a clear gap between the mature prostatic adenoma and the prostate capsule, and the crawling blood vessels of the prostate capsule are visible superficially, which facilitates precise haemostasis [16]. (2) There is no residual gland tissue following enucleation, which results in a smoother surgical wound and less bleeding. (3) A holmium laser can achieve point-to-point haemostasis. (4) The fossa is contracted when the bladder is empty, which facilitates precise haemostasis. (5) Urine has a procoagulant effect, and it comes into direct contact with the surgical wound in the prostatic fossa, promoting haemostasis [17].

This study discusses the strategy of no bladder irrigation after transurethral holmium laser prostate enucleation, which is an optimization of the transurethral holmium laser prostate enucleation surgical method. The primary drawback is the high learning curve associated with HoLEP [18, 19]. As a result, surgeons who do not perform bladder irrigation must be skilled in a variety of enucleation techniques. We do not advocate postoperative no-bladder irrigation to surgeons who are inexperienced with prostate enucleation surgery. In this study, the surgeon independently carried out more than 500 cases of HoLEP. Bleeding during prostate enucleation is a frequent and significant consequence, and patient safety must be protected. To ensure the safety of patients in Group A, we excluded patients with severe organ dysfunction, such as cardiovascular and cerebrovascular diseases, a prostate volume greater than 100 ml and other complicated conditions before surgery. We compared continuous bladder irrigation after HoLEP with postoperative no bladder irrigation, and the two groups of patients were considered equal in terms of functional outcomes. In addition, the patients who had an intraoperative perforation of the prostatic capsule had no obvious bleeding and were not treated specifically.

Within one month following surgery, there was no statistically significant difference in early postoperative complications between the two groups, such as urine retention, gross haematuria, haemorrhage, transitory urinary incontinence, urinary tract infection, testicular epididymitis, and urethral stricture. Before and one month after surgery, there were significant differences in IPSS, QoL, Qmax, and PVR in both groups. This is consistent with prior research results [20, 21]. At one month and six months following surgery, there was no statistically significant difference in IPSS, QoL, Qmax, or PVR between the two groups. This finding is also understandable because no bladder irrigation following HoLEP eliminates only one aspect of postoperative management. Both groups of patients essentially had identical surgical procedures. With the development of minimally invasive technology and the progress of medical devices, the strategy of no bladder irrigation after HoLEP will become a trend and this concept will also be accepted by more and more urologists.

Our present study has several limitations, including its retrospective nature and small sample size. Two groups of patients were not operated on concurrently, and additional prospective randomized comparative studies with long-term follow-up and larger cohorts are necessary to validate our findings. In addition, this study was not performed with other transurethral enucleations of the prostate to evaluate the effect of no-bladder irrigation. We will further design a large-sample prospective randomized controlled study to verify the safety of no bladder irrigation after HoLEP in the future. No bladder irrigation strategy combined with day-case HoLEP will further simplify treatment steps and reduce the catheterization time.

## Conclusion

The no bladder irrigation strategy after HoLEP is an improvement on the conventional surgical procedures for the treatment of BPH, which is safe and effective for appropriately selected patients according to the exclusion criteria.

## Data Availability

The datasets used and/or analysed during the current study available from the corresponding author on reasonable request.
